# *Lacticaseibacillus paracasei* PS23 Effectively Modulates Gut Microbiota Composition and Improves Gastrointestinal Function in Aged SAMP8 Mice

**DOI:** 10.3390/nu13041116

**Published:** 2021-03-29

**Authors:** Li-Han Chen, Ming-Fu Wang, Chun-Chao Chang, Shih-Yi Huang, Chun-Hsu Pan, Yao-Tsung Yeh, Cheng-Hsieh Huang, Ching-Hung Chan, Hui-Yu Huang

**Affiliations:** 1 Institute of Fisheries Science, National Taiwan University, Taipei 10617, Taiwan; lihan.h.chen@gmail.com; 2 Department of Life Science, National Taiwan University, Taipei 10617, Taiwan; 3 Department of Food and Nutrition, Providence University, Taichung 43301, Taiwan; mfwang@pu.edu.tw; 4 Division of Gastroenterology and Hepatology, Department of Internal Medicine, Taipei Medical University Hospital, Taipei 11031, Taiwan; Chunchao@tmu.edu.tw; 5 Division of Gastroenterology and Hepatology, Department of Internal Medicine, School of Medicine, College of Medicine, Taipei Medical University, Taipei 11031, Taiwan; 6 Graduate Institute of Metabolism and Obesity Sciences, Taipei Medical University, Taipei 11031, Taiwan; sihuang@tmu.edu.tw; 7 School of Pharmacy, Taipei Medical University, Taipei 11031, Taiwan; panch@tmu.edu.tw; 8 Aging and Disease Prevention Research Center, Fooyin University, Kaohsiung 83102, Taiwan; glycosamine@yahoo.com.tw (Y.-T.Y.); prevailingkimo@gmail.com (C.-H.H.); 9 Biomedical Analysis Center, Fooyin University Hospital, Pingtung 92849, Taiwan; 10 Department of Medical Laboratory Sciences and Biotechnology, Fooyin University, Kaohsiung 83102, Taiwan; 11 Program in Environmental and Occupational Medicine, Kaohsiung Medical University, Kaohsiung 80708, Taiwan; 12 Graduate Institute of Bioengineering, Tatung University, Taipei 10452, Taiwan; llfonly520@gmail.com

**Keywords:** *Lacticaseibacillus paracasei* PS23, lactobacillus, gut microbiota, gut function, age-related inflammation

## Abstract

Probiotics are reported to improve gastrointestinal (GI) function via regulating gut microbiota (GM). However, exactly how probiotics influence GM and GI function in elders is poorly characterized. Therefore, in this study, we assessed the effect of the probiotic *Lacticaseibacillus paracasei* PS23 (LPPS23) on the GM and GI function of aged mice. There were four groups of senescence-accelerated mouse prone-8 (SAMP8) mice (*n* = 4): a non-treated control group, a saline control group, a low dose LPPS23 group (1 × 10^8^ colony-forming unit (CFU)/mouse/day), and a high dose LPPS23 group (1 × 10^9^ CFU/mouse/day). Non-treated mice were euthanized at 16 weeks old, and others were euthanized at 28 weeks old. The next-generation sequencing results revealed that LPPS23 enriched *Lactobacillus* and *Candidatus_Saccharimonas*, while the abundance of *Lachnospiraceae*_UCG_001 decreased in aged mice given LPPS23. The abundance of *Lactobacillus* negatively correlated with the abundance of *Erysipelotrichaceae*. Moreover, LPPS23 improved the GI function of aged mice due to the longer intestine length, lower intestinal permeability, and higher phagocytosis in LPPS23-treated mice. The ELISA results showed that LPPS23 attenuated the alterations of pro-inflammatory factors and immunoglobulins. The abundance of LPPS23-enriched *Lactobacillus* was positively correlated with healthy GI function, while *Lachnospiraceae*_UCG_001, which was repressed by LPPS23, was negatively correlated with a healthy GI function in the aged mice according to Spearman’s correlation analysis. Taken together, LPPS23 can effectively modulate GM composition and improve GI function in aged SAMP8 mice.

## 1. Introduction

Aging is an important factor in several disorders that influence the quality of life of elderly individuals. The age-related alteration of gut microbiota (GM) composition is linked to age-related diseases, such as cognitive impairment, sarcopenia, and gastrointestinal (GI) dysfunction [1]. Therefore, good GM composition should prevent age-related disorders.

Several studies have addressed the interaction among GM, GI function, and aging. Sovran et al. linked GM alterations to age-associated impairments in GI mucus barrier function and age-related inflammation [2]. Moreover, aging significantly increases GI vulnerability, and 40% of geriatric patients report at least one GI complaint during routine physical examinations [3]. Previous studies have also indicated that age-related GM alterations and GI dysfunctions exist, such as bacterial overgrowth, increased intestinal permeability, and decreased absorption [4,5,6]. Furthermore, GM alterations and GI dysfunctions are suggested to cause age-related inflammation [7,8,9]. Thus, preventing age-related GI dysfunction should cooperate with GM to attenuate the diseases derived by age-related inflammation, such as sarcopenia, cognitive impairment, and metabolic diseases [10].

Probiotic supplementation is widely used to modulate GM composition and enhance GI function [11,12]. In addition, many probiotics have been reported to exert anti-inflammatory properties [13]. Therefore, probiotic supplementation may protect against GI dysfunction and inflammation in the elderly. However, to our knowledge, the precise effects of probiotics on GI function in the elderly remain unclear; only a small number of studies have investigated the effects of probiotic supplements in age-related disorders [14]. Thus, in order to provide a solid foundation for the use of probiotic-based strategies to prevent age-related diseases, it is important to investigate the effects of probiotics on GM and GI dysfunction in the aged subjects.

In our previous work, we demonstrated that probiotics could restructure GM and improve GI function. We also reported that *Lacticaseibacillus paracasei* PS23 (LPPS23) was beneficial in mitigating age-related diseases, including cognitive impairment and sarcopenia, in SAMP8 (senescence accelerated mouse prone-8) mice [14,15]. Moreover, administration of LPPS23 for 12 weeks significantly reduced age-related inflammation and protein uptake decline in SAMP8 mice [14]. Therefore, we assumed that LPPS23 could regulate the GM composition and GI function to prevent age-related disorders and so further investigated how LPPS23 influenced GM and GI function in the elderly by assessing the interactions between LPPS23 and the host. We administered LPPS23 to SAMP8 mice over the course of their lifespans, from youth into old age, and observed the effects of LPPS23 on GM composition and GI function.

## 2. Materials and Methods

### 2.1. Probiotics and Animals

LPPS23 was provided by Dr. Ying-Chieh Tsai (National Yang Ming Chiao Tung University, Taipei, Taiwan). LPPS23 is a strain isolated from healthy human feces and identified by phylogenetic classification of its 16S rRNA gene sequence. LPPS23 can induce anti-inflammatory effects and improve brain and muscle health [14,15,16]. In the present study, LPPS23 was subcultured before used. The doses of live LPPS23 used for administration were 1 × 10^8^ and 1 × 10^9^ colony-forming unit (CFU)/200 μL; SAMP8 mice were bred by Dr. Ming-Fu Wang. To make sure that age was the only factor to induce GI dysfunction, all mice were housed under standard laboratory conditions with a 12/12 h light/dark cycle at 22–24 °C and 40–60% humidity. SAMP8 mice were provided with a commercially available diet (local supplier) and sterile water ad libitum. Well-trained investigators monitored the conditions of the mice every day with no stress or discomfort observed in their behavior. Additionally, during the study, no unhealthy phenomena in the feeding conditions were observed.

The female mice were divided into four groups (*n* = 4): FC (16-week-old non-aged mice controls), FA (aged mice administered saline), FPS23L (aged mice administered low dose 1 × 10^8^ CFU LPPS23/mouse/day), and FPS23H (aged mice administered high dose 1 × 10^9^ LPPS23 CFU/mouse/day). The dose of LPPS23 was chosen based on our previous studies [14,15], which indicated LPPS23 at 1 × 10^9^ CFU/mouse/day prevented age-related decline in SAMP8 mice. The mice received 200 µL of saline or live LPPS23 at 9:00 a.m. by gavage every day for 12 weeks from 16 weeks of age until 28 weeks of age. The sample size was referred to in the previous studies regarding the composition of GM and gut function in mice [17,18].

The FC group was humanely sacrificed at 16 weeks old, and the other groups were sacrificed after 12 weeks of treatment at 28 weeks old. Intestine, intestinal mucosa, and fecal samples were collected, and intestinal length was measured. The mucosal samples were collected by intestine perfusion with 5 mL phosphate buffered saline (PBS) after sacrifice. Then, the supernatants were isolated by 2000× *g* centrifuge at 4 °C for 30 min for the analysis of pro-inflammatory factors and immunoglobulins. The animal protocol was approved by the Institutional Animal Care and Use Committee of Shih Chien University (IACUC-10407).

### 2.2. Intestinal Permeability

On the same day that the saline- or LPPS23-treated mice were to be euthanized, they were deprived of food 6 h prior to oral gavage of 200 μL of 80 mg/mL FITC–dextran (4 kDa; Sigma-Aldrich, St. Louis, MO, USA) followed by 4 h deprivation of both food and water. Blood samples were collected, and fluorescence intensity was measured on fluorescence plates using an excitation wavelength of 490 nm and an emission wavelength of 520 nm.

### 2.3. Enzyme-Linked Immunosorbent Assays

Pro-inflammatory factors in the intestinal mucosa were determined using sandwich ELISA (enzyme-linked immunosorbent assay) kits produced by Biolegend (San Diego, CA, USA) for tumor necrosis factor (TNF)-α and monocyte chemotactic protein (MCP)-1, according to the manufacturer’s instructions. The ELISA reader (BioTek, Winooski, VT, USA) was used to measure absorbance values of the samples.

### 2.4. Quantification of IgA, IgE, IgM, and IgG in the Intestinal Mucosa

The levels of IgA, IgE, IgM, and IgG in the intestinal mucosa were analyzed using a mouse IgA ELISA kit (eBioscience, Santa Clara, CA, USA), mouse IgE ELISA kit (Biolegend), mouse IgM ELISA kit (Biolegend), and mouse Total IgG ELISA kit (eBioscience) according to the manufacturers’ instructions. The plates were read at 450 nm.

### 2.5. Bacterial Genomic DNA Isolation

Total genomic DNA was isolated from 200 mg fecal samples using the QIAamp DNA Stool Mini kit (Qiagen, Hilden, Germany) according to the manufacturer’s instructions. The concentrations of DNA were measured with a NanoDrop2000 (Thermo Scientific, Waltham, MA, USA), and the samples were stored at −80 °C until use.

### 2.6. 16S rRNA Gene Sequencing and Data Analysis

The V3-V4 region of the 16S rRNA gene was PCR-amplified using the primers 341F (5′-CCTAYGGGRBGCASCAG-3′) and 806R (5′-GGACTACNNGGGTATCTAAT-3′) to develop the amplicon libraries according to recommended Illumina 16S Metagenomic Sequencing Library Preparation manual protocol. Then, the amplicons were paired-end sequenced (PE 2 × 250) using an Illumina HiSeq 2000 platform according to the manufacturer’s protocol. The paired forward and reverse reads passed quality control and were merged, and then mapped to the Silva database to construct operational taxonomic units (OTUs) at 97% identity through the UPARSE pipeline (drive5, Tiburon, CA, USA) [19].

Quantitative Insights Into Microbial Ecology (QIIME) [20] was used to analyze data. Chimeric sequences were removed using ChimeraSlayer [21]. Sequences with ≥97% similarity were assigned to the same operational taxonomic units (OTUs). The Silva database [22] was used to annotate the genes. Alpha diversity analysis (Shannon index) was assessed using QIIME. Beta diversity was analyzed using weighted principal coordinate analysis (PCoA) and partial least squares discriminant analysis (PLS-DA) using QIIME; PERMANOVA was used for analyzing statistical significance. Linear discriminant analysis (LDA) effect size (LefSe) was performed online using the Galaxy workflow framework [23].

### 2.7. Statistical Analyses

Data are presented as the mean ± standard error of the mean (SEM). Data of bacteria were analyzed using nonparametric one-way ANOVA with one-way ANOVA post hoc, and the other data were analyzed using one-way ANOVA with a Tukey honestly significant difference (HSD) post hoc test. Rank tests with Spearman’s correlation coefficient were used to assess associations between two bacteria, between bacteria and parameters of intestinal function, between bacteria and pro-inflammatory factors, and between bacteria and Ig. A *p*-value < 0.05 was considered statistically significant.

## 3. Results

### 3.1. Effects of LPPS23 on GM Community Composition and Diversity

**Figure 1:**
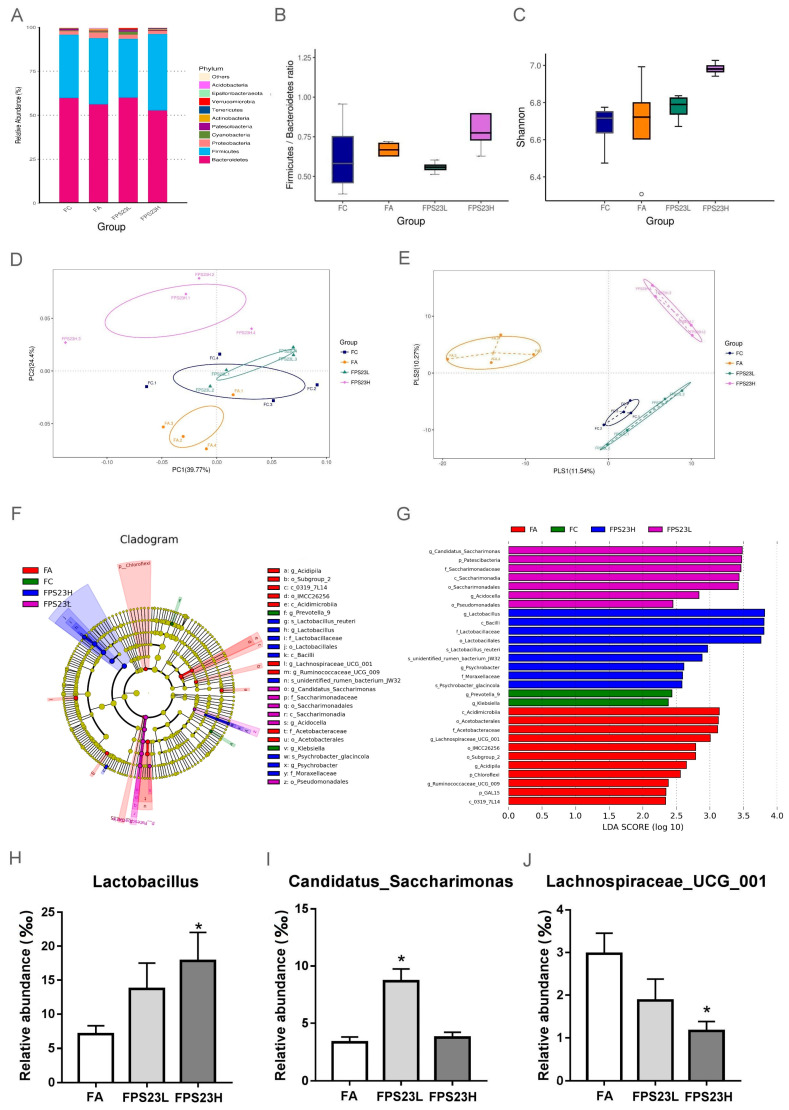
Gut microbiota composition. (**A**) Bacterial community distribution; (**B**) ratio of Firmicutes/Bacteroidetes; (**C**) alpha diversity indicated by Shannon’s diversity index; (**D**,**E**) beta diversity indicated by weighted principal co-ordinates analysis (PCoA) (**D**) and partial least squares discriminant analysis (PLS-DA) (**E**) in senescence accelerated mouse prone-8 (SAMP8) mice; (**F**,**G**) Linear discriminant analysis effect size (LEfSe) analysis of the gut microbiota of SAMP8 mice; (**H**–**J**) relative abundance of *Lactobacillus* (**H**), *Candidatus_Saccharimonas* (**I**), and *Lachnospiraceae* UCG 001 (**J**) in the aged SAMP8 mice. Asterisk (*) indicates significant differences from the female aged mice administered saline (FA) (*p* < 0.05). *n* = 4.

The three major bacterial phyla identified in the SAMP8 mice feces were *Bacteroidetes*, *Firmicutes*, and *Proteobacteria* (Figure 1A), which were evaluated by at least 52,597 reads/sample. Moreover, the ratio of Firmicutes/Bacteroidetes, an indicator of gut inflammation [24], was no different among the groups (Figure 1B). Alpha diversity was not significantly different between the FC, FA, FPS23L, and FPS23H groups (Figure 1C). The microbial communities of all groups were separated by the principal co-ordinates analysis (PCoA) (Figure 1D) and partial least squares–discriminant analysis (PLSDA) (*p* = 0.042) (Figure 1E), though there was no statistical difference in the PCoA analysis (*p* = 0.153).

### 3.2. Effects of LPPS23 on Abundant GM

Next, we identified the microbial species that were enriched from the class to species level using LEfSe analysis. Bacteria belonging to the order *Lactobacillales* increased in the FPS23H mice. Moreover, both the high and low doses of LPPS23 enhanced bacteria belonging to the order *Pseudomonadales*, and the low dose of LPPS23 promoted bacteria from the class *Saccharimonadia*. The bacteria enriched in the FA group belonged to the class *Acidimicrobiia*, genus *Lachnospiraceae*_UCG_001, genus *Ruminococcaceae*_ UCG_009, and order *Acetobacterales*. Bacteria from the genus *Prevotella* and *Klebsiella* were enriched in the FC group (Figure 1F,G).

Bacteria with LDA scores > 3.0 at the genus level in the aged mice were also compared between the LPPS23-treated and aged control mice. *Lactobacillus* (Figure 1H) was significantly enriched in the FPS23H mice, and the enrichment of *Candidatus_Saccharimonas* (Figure 1I) was observed in the FPS23L mice. Moreover, *Lachnospiraceae_UCG_001* (Figure 1J) abundance decreased in FPS23H mice.

### 3.3. Bacteria Correlated with Lactobacillus

**Figure 2:**
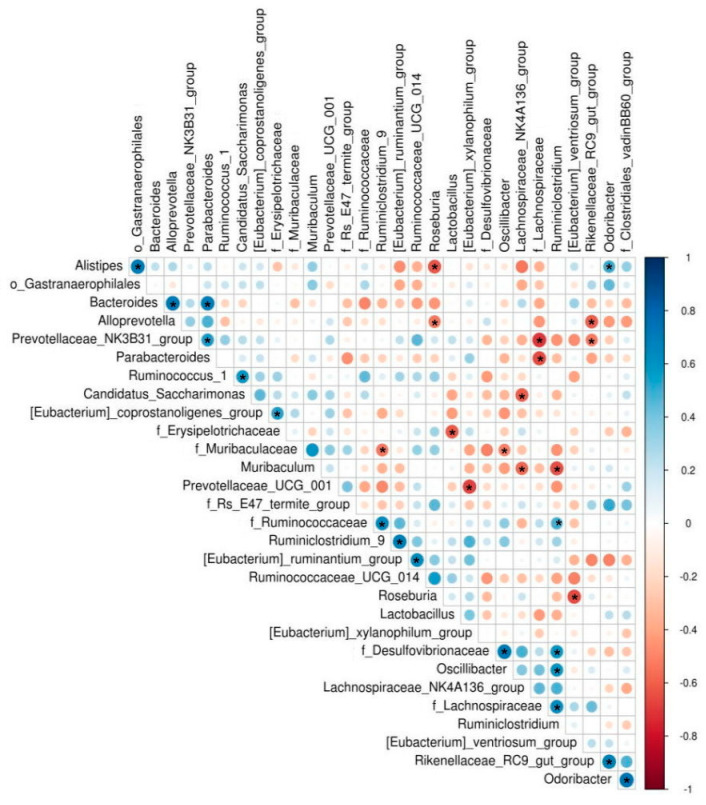
Spearman’s correlation analysis of the gut microbiota of SAMP8 mice. Asterisk (*) indicates *p* < 0.05. *n* = 4.

We used Spearman’s correlation analysis to identify bacteria that are correlated with *Lactobacillus*. *Lactobacillus* was negatively correlated with bacteria from the family *Erysipelotrichaceae* in mice (*r* = −0.62, *p* = 0.035) (Figure 2).

### 3.4. Effects of LPPS23 on Intestinal Parameters

**Figure 3:**
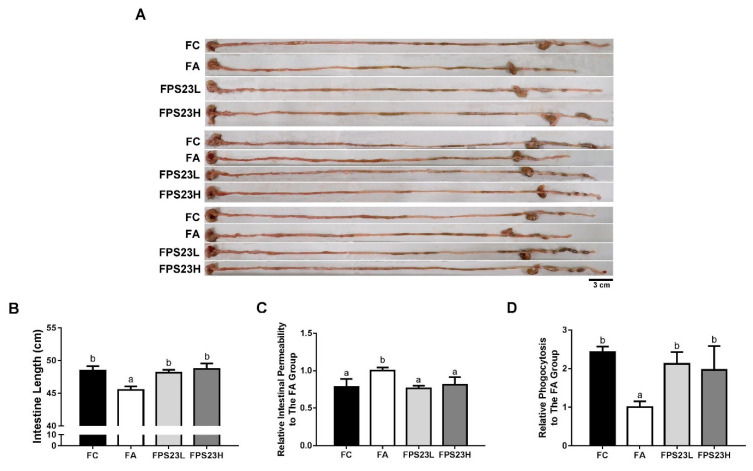
Intestinal function. (**A**) Representative experiments of intestine length, (**B**) intestinal length, (**C**) intestinal permeability, and (**D**) phagocytosis in the SAMP8 mice. Different superscript letters (a, b, c) indicate significant differences in one-way ANOVA by Tukey’s honestly significant difference (HSD) post hoc test (*p* < 0.05). *n* = 4. Scale bar = 3 cm.

Since LPPS23 regulated GM composition in the present study and was reported to enhance the protein uptake in the aged SAMP8 mice [14], LPPS23 might have effects on GI function. As age-related intestinal shortening and barrier dysfunction are associated with GM alterations [25], we measured intestinal length, intestinal permeability, and phagocytotic activity in 16-week-old untreated SAMP8 mice and 28-week-old SAMP8 mice after administration of saline or LPPS23 daily for 12 weeks to assess the effects of LPPS23 on the age-related change in the intestine. Significant age-related declines in intestinal function were observed in the mice including decreased length of the intestine (Figure 3A,B), increased intestinal permeability (Figure 3C), and decreased phagocytotic activity (Figure 3D) in the FA group compared to the FC group. Moreover, LPPS23 treatment prevented these age-related changes in the mice. These results indicated that LPPS23 might maintain intestinal function during the aging process.

### 3.5. Effects of LPPS23 on Levels of the Pro-Inflammatory Factors TNF-α and MCP-1

**Figure 4:**
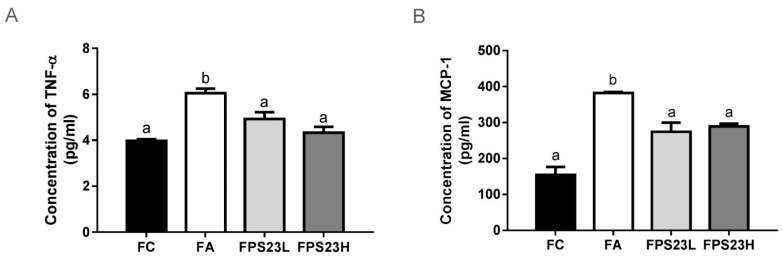

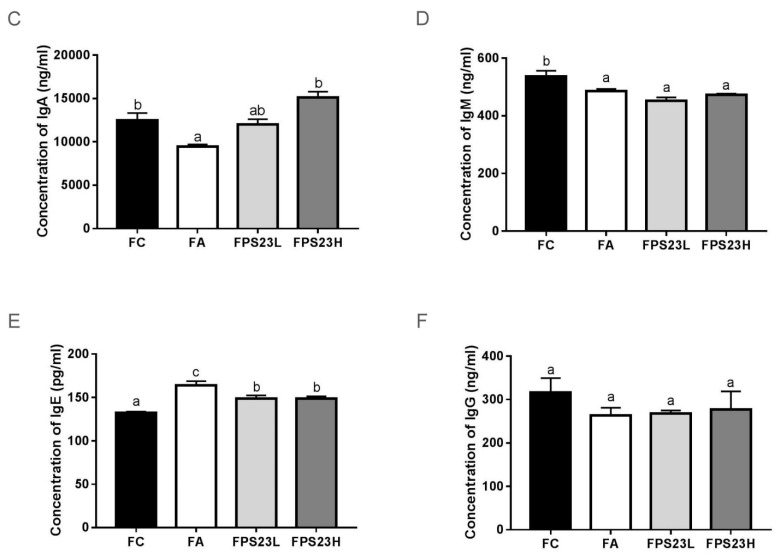
Pro-inflammatory cytokines and immunoglobulins in the intestinal mucosa. (**A**) TNF-α; (**B**) MCP-1; (**C**) IgA; (**D**) IgM; (**E**) IgE; (**F**) IgG in the SAMP8 mice. Different superscript letters (a, b, c) indicate significant differences in one-way ANOVA by Tukey HSD post-hoc test (*p* < 0.05). *n* = 4.

Levels of the pro-inflammatory factors TNF-α and MCP-1 were quantified to assess the effect of LPPS23 on the aging intestine. Both pro-inflammatory factors increased during aging. LPPS23 suppressed these age-related changes in mice (Figure 4A,B). However, there was no dose response effect for LPPS23 (Figure 4A,B).

### 3.6. Effects of LPPS23 on Levels of Immunoglobulins in the Intestinal Mucosa

The levels of immunoglobulins in the intestinal mucosa were also quantified to investigate immune function. Aging reduced the levels of IgA (Figure 4C) and IgM (Figure 4D), induced IgE levels (Figure 4E), and had no impact on IgG (Figure 4F) in mice. Moreover, these age-related changes in IgA (Figure 4C) and IgE (Figure 4E) in mice were significantly reduced by the administration of LPPS23. The higher dose of LPPS23 greatly reduced the age-related decline of IgA more than the lower dose of LPPS23.

### 3.7. Correlation of LPPS23-Modulated Bacteria and GI Conditions

**Figure 5:**
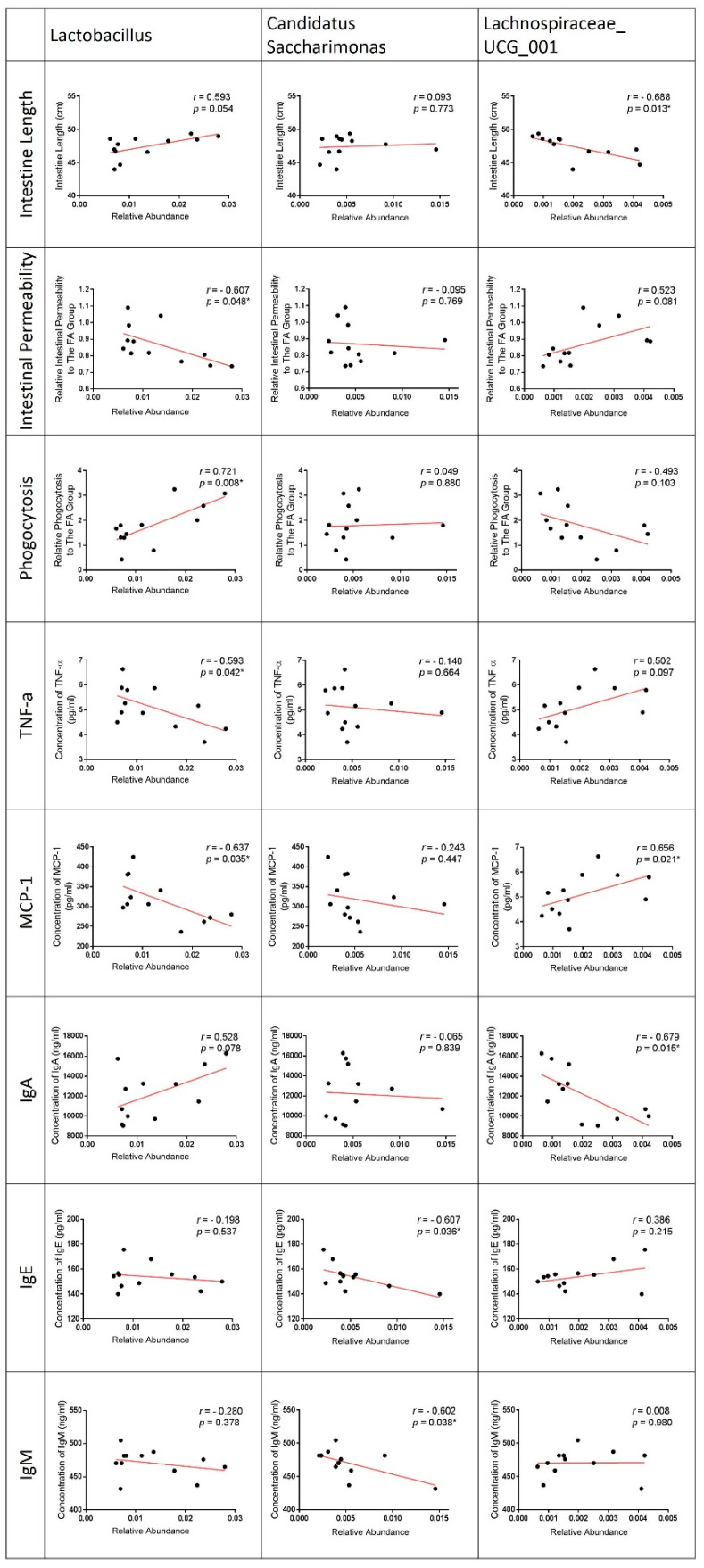
Correlation of *Lacticaseibacillus Paracasei* PS23 (LPPS23)-modulated bacteria and gastrointestinal (GI) condition. Rank tests with Spearman’s correlation coefficient were used to assess correlations between bacteria (*Lactobacillus*, *Candidatus_Saccharimonas*, and *Lachnospiraceae*_UCG_ 001) and parameters of intestinal function (intestine length, intestinal permeability, and phagocytosis), pro-inflammatory factors (TNF-α and MCP-1), and immunoglobulin (IgA, IgE, and IgM). Asterisk (*) indicates *p* < 0.05. *n* = 12.

The correlation of bacteria and GI conditions was analyzed, because LPPS23 significantly altered GI conditions and the abundance of *Lactobacillus*, *Candidatus_Saccharimonas*, and *Lachnospiraceae*_UCG_001 (Figure 5) in aged SAMP8 mice. The abundance of *Lactobacillus* was positively correlated with the intestine length and phagocytotic activity, while a negative correlation was observed between *Lactobacillus* and the intestinal permeability and concentration of the pro-inflammatory factors, TNF-α and MCP-1. *Candidatus_Saccharimonas* (which was significantly increased in the FPS23L group) was negatively correlated with the concentration of mucosal IgE and IgM. *Lachnospiraceae*_UCG_001 (which was reduced by the high dose of LPPS23) was negatively correlated with the intestine length and concentration of IgA, while it was positively correlated with the concentration of MCP-1 (Figure 5). The trends of correlation with the GI conditions were similar in *Lactobacillus* and *Candidatus_Saccharimonas*, but that of *Lachnospiraceae*_UCG_001 was different from the others (Figure 5).

## 4. Discussion

The number of individuals with age-related disorders is increasing due to longer life spans. Our previous studies reported that LPPS23 attenuated the age-related cognitive impairment, muscle loss, and protein uptake decline in SAMP8 mice [14,15], and these age-related problems were associated with the change in GM composition [26,27]. Therefore, we attempted to understand the interaction of LPPS23 and GM in the aged SAMP8 mice. Although the probiotic did not influence the alpha diversity of the GM in the mice, high-dose LPPS23 (10^9^ CFU/mL per day for 12 weeks) altered beta diversity and increased the abundance of *Lactobacillus* in the GM of the mice. Moreover, a negative correlation was observed between *Lactobacillus* and *Erysipelotrichaceae.* Furthermore, we identified three bacterial genera that were significantly regulated by LPPS23 in the aged mice: *Lactobacillus*, *Candidatus_Saccharimonas*, and *Lachnospiraceae_UCG_001*.

Due to the bidirectional effect of GM composition and GI function, the GI function was further investigated in SAMP8 mice. We observed severe intestinal dysfunction associated with inflammation and changes in IgA, IgE, and IgM concentrations in the intestinal mucosa of aged SAMP8 mice. These age-related alterations were alleviated by the administration of LPPS23 and were significantly correlated with the aforementioned LPPS23-modulated bacterial genera. Thus, we have provided the first demonstration of a relationship among probiotics, GM, and aging GI tracts.

Probiotics are thought to influence GI health by regulating the composition of the GM [28]. Several species and strains of *Lactobacilli* have been reported to change the population of bacteria in the GM, including *Lactobacillus casei*, *Lactobacillus acidophilus*, *Lactobacillus helveticus*, and *Lactobacillus rhamnosus* [29]. Similarly, we found that LPPS23 altered the beta-diversity of the GM and increased the abundance of *Lactobacillus*. Enrichment of *Lactobacillus* has been linked to healthy intestines and reduced inflammation [30,31]. Our results also demonstrated a positive correlation between the abundance of *Lactobacillus* and factors of GI health, such as a longer intestine, lower intestinal permeability, and higher phagocytosis. Moreover, the FPS23H mice exhibited the highest abundance of *Lactocacillus* and the best intestinal function among the aged mice groups. Taken together, the results suggest that LPPS23 attenuates the GI dysfunction in aged SAMP8 mice by regulating the GM in ways such as the enrichment of *Lactobacillus*.

GI dysfunction and inflammation induce each other. Therefore, several probiotics belonging to *Lactobacillus* have been reported to improve both GI function and inflammation. For example, Oliveira et al. (2018) found that *Lactobacillus rhamnosus* ST11 reduces intestinal inflammation and GI dysfunction in an adoptive transfer mouse model of experimental colitis [32]. Another study demonstrated that *Lactobacillus acidophilus* NCFM attenuates TNF-α-induced intestinal dysfunction and inflammation [33]. Our previous study also revealed that LPPS23 improved the intestinal function in protein uptake and prevented the increase in pro-inflammatory factors in the serum, muscle, and brain of aged SAMP8 mice [14,15]. Thus, this raises the question of whether *Lactobacillus* can also improve GI dysfunction and age-related inflammation in the elderly. The results of this study revealed that LPPS23 increased the abundance of *Lactobacillus*, which was negatively correlated with the concentration of pro-inflammatory factors TNF-α and MCP-1. Therefore, the results suggest LPPS23 could prevent not only intestinal function but also age-related inflammation by regulating the GM in the aged mice.

Since LPPS23 increased *Lactobacillus* that benefited intestinal function [34], we examined the correlations between *Lactobacillus* and other bacterial species. Only *Erysipelotrichaceae* was correlated negatively with *Lactobacillus*. *Erysipelotrichaceae* was enriched in a colorectal cancer mouse model [35] and in mice exhibiting colitis after treatment with dextran sodium sulphate [36]. Moreover, Thevaranjan et al. (2017) demonstrated that *Erysipelotrichaceae* was increased in aged mice [37]. Therefore, LPPS23 may cause increased *Lactobacillus* abundance resulting in decreased *Erysipelotrichaceae* abundance, which in turn may maintain or improve intestinal function and reduce inflammation in aged mice.

In addition to *Lactobacillus*, the relative abundances of other bacteria were changed in SAMP8 mice treated either with a high or low dose of LPPS23. All of the significantly changed bacteria (LDA score > 3.0 and *p* < 0.05) at the genus level were related to intestinal inflammation. *Lachnospiraceae*_UCG_001, a bacterium found to be increased in feces from unhealthy inflamed intestines [38], was reduced in the aged mice treated with the high dose of LPPS23. The relationship between *Lachnospiraceae*_UCG_001 and unhealthy intestines was also confirmed in the present study by the negative correlation between the abundance of *Lachnospiraceae*_UCG_001 and health factors, including longer intestines, a higher concentration of IgA, and a lower concentration of MCP-1. Furthermore, *Candidatus_Saccharimonas*, which was negatively correlated with intestinal inflammation [39,40], was more abundant in the FPS23L than FA mice. Our results also indicated a negative correlation between *Candidatus_Saccharimonas and IgE*, which is an immunoglobulin that increases because of immunosenescence and GI dysfunction resulting in age-related chronic *inflammation* [41,42,43]. Therefore, LPPS23 might improve the GI function in the aged SAMP8 mice through establishing an anti-inflammatory microenvironment in the intestine to prevent GI dysfunction caused by age-related inflammation. As such, we were able to determine potential bacterial biomarkers (i.e., *Erysipelotrichaceae, Lachnospiraceae*_UCG_001, and *Candidatus_saccharimonas*) of intestinal inflammation and GI function in the aged population.

Because only live LPPS23 was investigated in the present study, it raises the question of whether the effects of LPPS23 were caused when it was passing through or colonizing the intestine. According to the Liao et al. study, both dead and live LPPS23 showed similar abilities in the reduction of inflammation [16]. Therefore, establishing itself in the gut may not be a requirement for LPPS23 to achieve its effect in the present study. However, aged mice were used in the present study, while Liao et al. used young mice. Thus, further study is necessary to understand whether live LPPS23 is required to obtain the results of the present study.

The sample size of this study was referenced from previous studies regarding GM composition and GI function in mice [17,18]. Moreover, our results demonstrate that LPPS23 significantly improved GI function and changed the properties of three bacteria at the genus level, especially *Lactobacillus* (*p* < 0.05). The statistical powers were also calculated and were higher than 0.8 in the results of GI function and condition with a significant difference. Thus, the animal count should be reasonable and provide enough statistical significance in the present study. Furthermore, the anti-inflammatory effects of LPPS23 were demonstrated not only in the SAMP8 mice studies [14,15] but also in the C57BL/6J mice studies [16,44]. These studies also suggested that LPPS23 might influence gut microbiota and condition, because the effects of LPPS23 seemed to be implemented either via the gut–brain or gut–muscle axes. Since the present study showed that LPPS23 could prevent inflammation, regulate gut microbiota, and maintain intestine function, the results of the present study should be general and representative. However, it cannot be excluded that performing the study with more samples in each group might reveal more information. Therefore, increasing the sample number will be considered in further research.

SAMP8 mice are used most often for age-related neuropathological research, but more and more studies show that SAMP8 mice are also an optimal model for age-related disorders, such as muscle and mitochondria impairments [14]. The previous studies revealed acceleration in the appearance of senescence and age-related decrease in protein uptake in SAMP8 mice [14,15,45]. In the present study, 28-week-old SAMP8 mice showed several changes in phenomena that were related to aging intestines in rodents and humans. For example, increases in intestinal permeability and pro-inflammatory cytokines are observed in both aged rodents and humans [4,5,46,47]. Therefore, accelerated senescence seems to occur systemically in SAMP8 mice, which should be an appropriate model for intestinal age-related studies. It is unsurprising that SAMP8 mice were utilized for studying GI function by different scientists [48,49,50]. However, there were still some phenomena related to aging intestines in humans not assessed in the previous and present studies. Therefore, more investigations are necessary to evaluate how well SAMP8 mice can represent humans in aged-related GI function studies.

Although the results of the present study strongly supported the LPPS23 effectively modulating gut microbiota composition and improving gastrointestinal function in aged SAMP8 mice, there were limitations. First, the sample number was relatively small in the present study. The study was performed with well-trained investigators, inbred strain mice from the same batch, and in a well-controlled environment to lower the variation. Therefore, significant differences were revealed between the groups. However, to increase the samples, we may need more information to understand the effect of LPPS23 on microbiota and GI condition. Second, the effect of LPPS23 was observed in mice, not humans. Thus, a clinical trial will be necessary to confirm the function of LPPS23 in the future.

In conclusion, we have provided the first piece of evidence that LPPS23 can prevent age-related intestinal dysfunction and inflammation by modulating the GM. LPPS23 supplementation merits further research as a potential strategy to maintain GI health in elderly individuals.

## Data Availability

The data in this study are available on request from the corresponding author.
